# Calcium phosphate cement promotes the stability of osteoporotic lumbar pedicle screw by enhancer-injecters with different number of holes

**DOI:** 10.1186/s12893-023-02235-9

**Published:** 2023-11-18

**Authors:** Suochao Fu, Yu Zhang, Renkai Wang, Xiaobao Zou, Fuzhi Ai, Jianhua Wang, Xiangyang Ma, Hong Xia, Wei Lei

**Affiliations:** 1 Department of Orthopedics, General Hospital of Southern Theater Command of PLA, Guangzhou, 510000 People’s Republic of China; 2 Department of Orthopedics, Sun Yat-Sen Memorial Hospital, Sun Yat-Sen University, Guangzhou, 510020 People’s Republic of China; 3 Fourth Department of Orthopedics, Xijing Hospital, Air Force Military Medical University, Xi’an, 733399 People’s Republic of China

**Keywords:** Calcium phosphate cement, Enhancer-injecter, Lumbar pedicle screw, Osteoporosis, Stability

## Abstract

**Backgrounds:**

This study aimed to compare whether Calcium phosphate cement (CPC) promotes the stability of osteoporotic lumbar pedicle screw by enhancer-injecters with different number of holes.

**Methods:**

Through a self-designed bone cement injection device, the pedicle screw canal was strengthened with calcium phosphate bone cement, and divided into 4-hole group, 6-hole group, 8-hole group, straight pore group and the control group. The screw was inserted into the mechanical test module, the Maximum insertion torque and Maximum axial pull-out strength were recorded, and the distribution of calcium phosphate bone cement was analyzed by CT and X-ray. The data results were analyzed using SPSS19.0 statistical software package.

**Results:**

The distribution of bone cement in different reinforcement groups was different and showed regularity. The bone cement in the 4-hole group was roughly located in the head 1/3 of the screw, the 6-hole group was located in the middle 1/3 of the screw, and the 8-hole group was located in the caudal 1/3 of the screw. Compared with the control group, the maximum axial pull-out force of screws in the lateral hole and full screw tunnel reinforcement group was significantly increased. There was no significant difference between the 4-hole, 6-hole and straight pore groups. There was no difference in the screw-in torque between the reinforcement groups, and they all increased significantly compared with the control group, and the difference was statistically significant. After the screw was pulled out, the interface between the bone cement and the polyurethane material was fractured, and a tight package was formed with the screw.

**Conclusions:**

Enhancer syringes with different hole numbers combined with CPC bone cement injection can significantly increase the maximum screw pull-out force. The 8-hole group has a smaller pull-out force and is relatively prone to leakage of reinforcing material, which lacks safety in use. The local reinforcement of 4-hole and 6-hole sheath can play a similar role to that of total nail tunnel reinforcement.

## Introduction

Vertebral compression fractures (VCF) are one of the most common fragile fractures and often require internal fixation [1]. Pedicle screws are commonly used for internal fixation. It has been widely used because of its realization of 3D fixation and its convenience in short segment internal fixation. However, due to poor bone condition and lack of good bone bed, pedicle screw internal fixation in patients with osteoporosis often leads to serious complications such as loosening and release of internal fixation screws, and even loss of the opportunity for internal fixation [2]. Thus, It is necessary to strengthen the osteoporotic vertebral body with internal fixation.

Since the chemical composition of calcium phosphate cement is the same as that of bone mineral [3], it is often used to repair bone defects and increase the stability of pedicle screw canal [4]. The pedicle screw canal was filled with bone cement, and then the screw was placed. It was found that it could effectively increase the axial extraction force of the pedicle screw. It has also been confirmed that CPC is beneficial to the fixation of pedicle screws under the condition of osteoporosis and can play a strengthening role. Because CPC can induce biodegradable bone, it can promote the formation of new bone while degrading, and it is non-toxic and has no obvious exothermic characteristics. Furthermore, CPC can enhance its physical, mechanical, and biological properties by adding biomaterials to its composition. This allows it to be increasingly used in the field of spinal surgery [5].

In our study, previously designed enhancer-injecters with different number of holes were used to verify the effect of CPC injection on the augmentation of pedicle screw in the Chinese osteoporotic lumbar pedicle channel through biomechanical analysis [6].

## Methods and materials

### Design of study

In this study, we used the biomechanical test module (Pacifc Research Laboratory Corp, USA) synthesized by polyurethane material, which had the characteristics of isotropy and homogeneity. Furthermore, this module contains more than 95% open pore structure with an average pore size of 1.5 to 2.5 mm, simulating the cellular structure of cancellous bone under osteoporosis and suitable for penetration of injectable bone cement materials.

In this study, we performed biomechanical experiments based on the three groups, including a local augmentation group, a full-length augmentation group, and a control group. We designed the hole of the local augmentation group to be located on the side wall of the sheath, which including 4-hole group, 6-hole group and 8-hole group. Then We designed a full-length augmentation group consisting of injectors with straight pore but no side holes. The design of enhancer injecter, bone cement injector, straight pore design can be seen in our previous study [6].

### The experimental group

According to the difference of whether bone cement was injected or not and the number of holes in the syringe with fortification agent, they were divided into 5 groups, namely, 4-hole group, 6-hole group, 8-hole group, straight pore group and the control group. The sample size of each group was 6.

### Pedicle screw path preparation

The center point was selected according to the plane of PU module 13 cm×4 cm, and the pedicle screw path was prepared conventionally. Use a 3.5 mm diameter hand drill to carefully place holes on the module. Ensure that the drill bit is always perpendicular to the module. The entry depth is 45 mm. During the process, try to avoid the shaking of the hand drill and the expansion of the nail path. Place holes in all nail paths in turn.

### Mixing and filling of calcium phosphate bone cement

We made the calcium phosphate bone cement (CPC) into a paste according to the protocols. The CPC was produced by Shanghai rebone Biological, a mixture of 3.8 g powder and 1.85ml water. For the perfusion of CPC, the lateral foramen sheath inserted into the nail canal was inserted at the same depth to 45 mm, and the CPC was pushed into the lateral foramen sheath at a constant speed. The sheath is then left still, unattached to the syringe, and the pushrod is quickly inserted into the sheath. The remaining CPC in the sheath was completely perfused into the material surrounding the nail path.

### Biomechanical analysis

#### Maximum insertion torque

We used a torque wrench to make a stable connection to the pedicle screw entrer. Screw the screw into the mechanical test module at 3 RPM according to ASTM F 543-02. And record the maximum torque value.

#### Maximum axial pull-out strength

The pedicle screw extraction force test was performed at a speed of 5 mm/min according to ASTM F 543-02. When the screw is pulled out after the module is damaged, it is defined as the maximum axial pulling force of the screw.

### Data analysis

SPSS19.0 statistical software was used for statistical analysis. The maximum screw torque and maximum axial pulling force were expressed by measurement data (mean ± standard deviation) and one-way analysis of variance was used. SNK-q test was used for comparison between groups, and P < 0.05 meant the difference was statistically significant.

## Results

### Biomechanical analysis

**Fig. 1:**
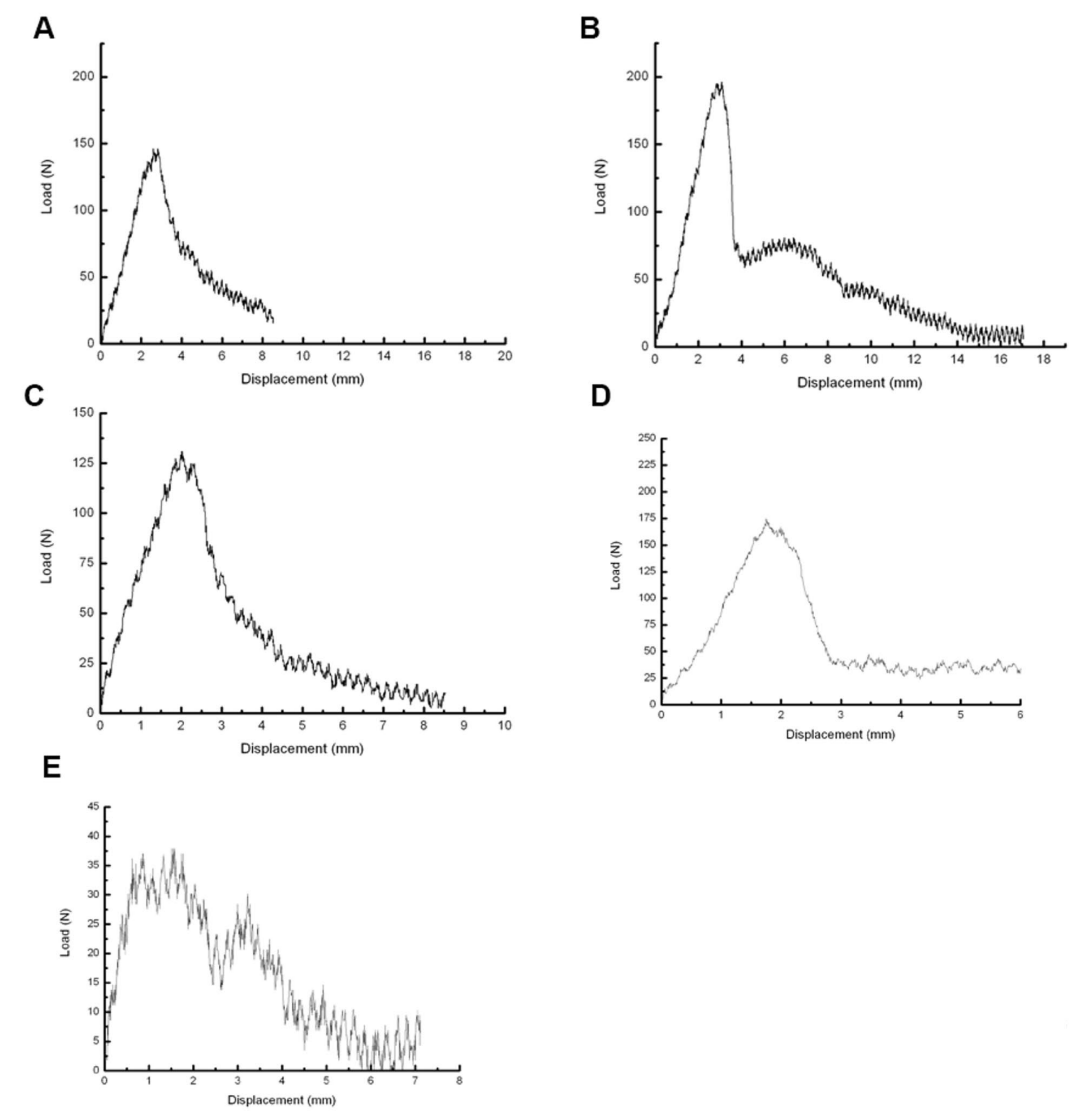
Loading displacement curves. **(A)** The 4-hole group; **(B)** The 6-hole group; **(C)** The 8-hole group; **(D)** The straight pore group; **(E)** The control group

The SNK-q test showed that the maximum torque of 4-holes, 6-holes, 8-holes and straight pore groups was significantly different from that of control group (P < 0.01). There was no significant difference in the torque between the groups 4-holes, 6-holes, 8-holes and straight pore group (P > 0.05). The comparison of screw extraction forces among different groups was conducted by one-way analysis of variance and SNK-q test. The results showed that the 4-holes, 6-holes groups and straight pore group were higher than the 8 well group, and the differences were statistically significant (P < 0.01). However, there was no significant difference in pairwise comparison among all groups in 4-holes, 6-holes and straight pore groups (P > 0.05). There were significant differences between all groups and control group (P < 0.01) (Table 1). The results showed that the screw extraction force increased significantly after CPC bone cement injection. The injection of 4, 6 and the straight pore channel had a better effect on the extraction force. Furthermore, we showed the loading displacement curves in the Fig. 1.

**Table 1:** The maximum insertion torque and maximum axial pull-out strength

Value	4 holes	6 holes	8 holes	Straight pore	control	P
maximum insertion torque	0.11 ± 0.01	0.12 ± 0.01	0.11 ± 0.01	0.10 ± 0.01	0.06 ± 0.01	< 0.01
maximum axial pull-out strength	162.93 ± 29.73	174.86 ± 35.29	115.98 ± 14.41	171.83 ± 16.07	40.92 ± 6.73	< 0.01

### Distribution of CPC and screw -CPC-PU interface observation

**Fig. 2:**
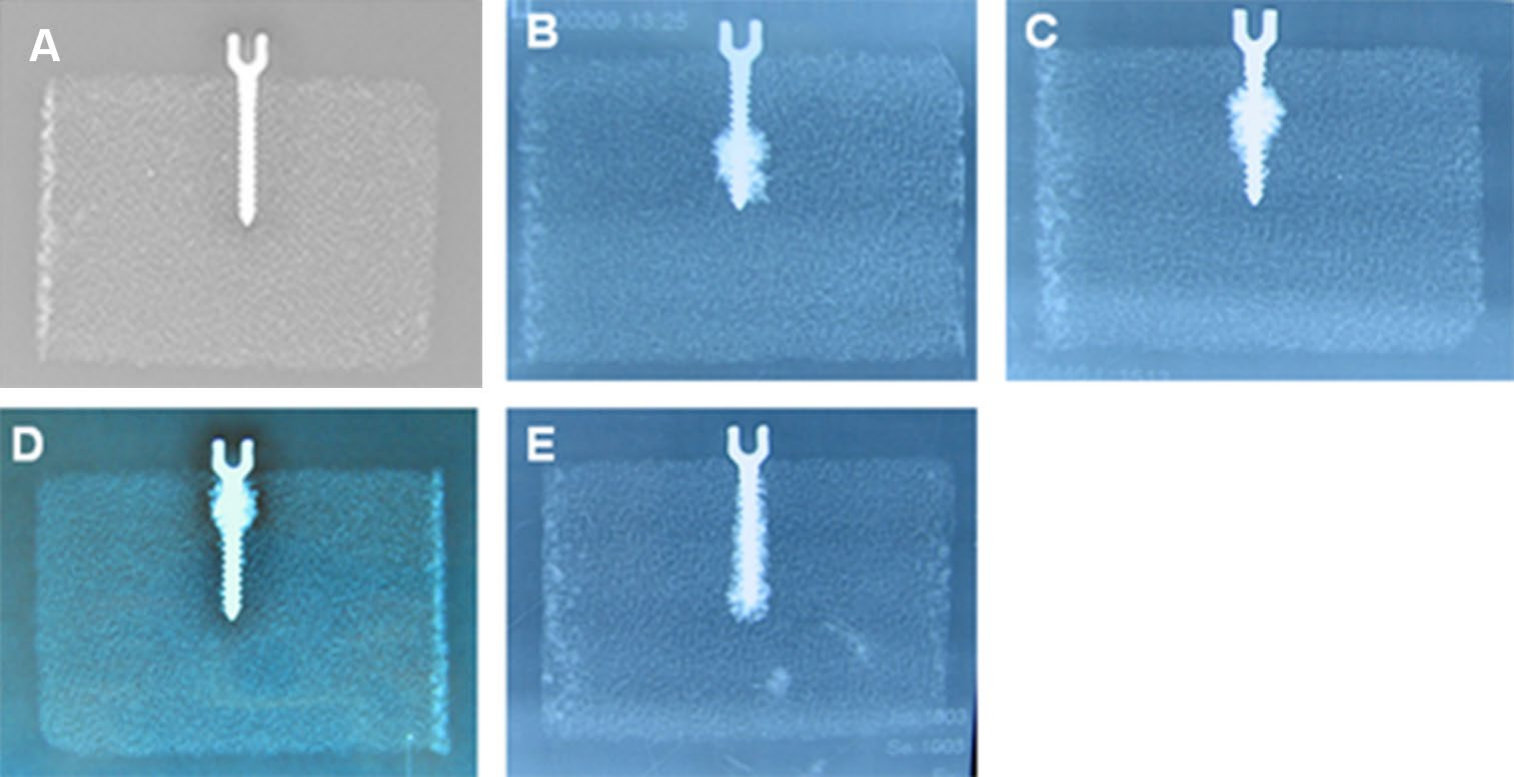
The distribution of bone cement in module based on X-ray. **(A)** Anterior posterior view of the control group; **(B)** Anterior posterior view of the 4-hole group; **(C)** Anterior posterior view of the 6-hole group; **(D)** Anterior posterior view of the 8-hole group; **(E)** Anterior posterior view of the straight pore group

**Fig. 3:**
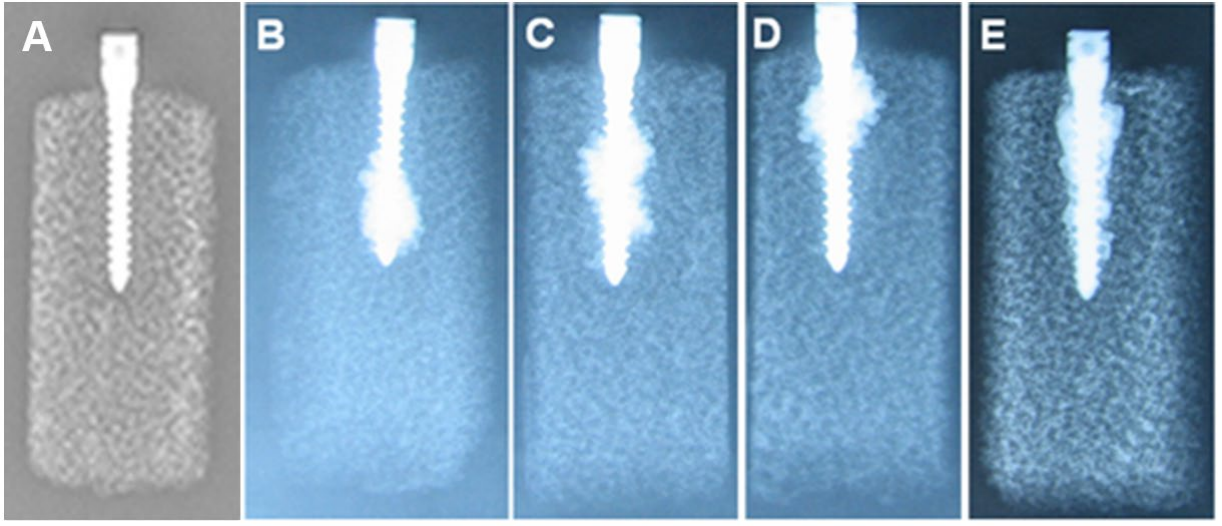
The distribution of bone cement in module based on X-ray. **(A)** Lateral view of the control group; **(B)** Lateral view of the 4-hole group; **(C)** Lateral view of the 6-hole group; **(D)** Lateral view of the 8-hole group; **(E)** Lateral view of the straight pore group

**Fig. 4:**
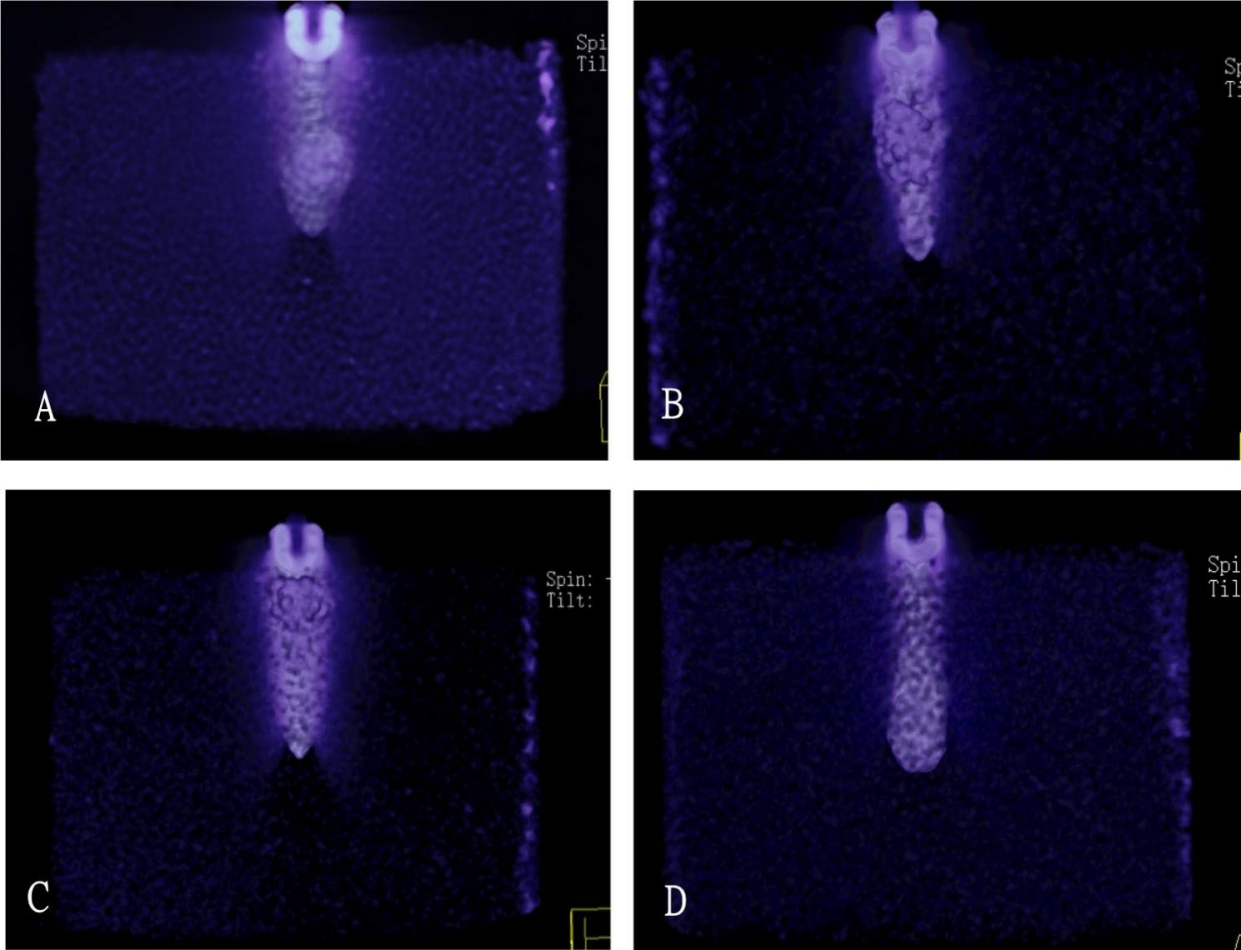
The distribution of bone cement in module based on CT. **(A)** CT scan of the 4-hole group; **(B)** CT scan of the 6-hole group; **(C)** CT scan of the 8-hole group; **(D)** CT scan of the straight pore group

The calcium phosphate bone cement also showed a certain distribution regularity when it combined with the pedicle screw. The bone cement in the 4-holes group was mainly distributed in the distal end of the screw, showing a spiral trend. The bone cement in the 6-holes group was mainly distributed in the far middle of the screw, and the bone cement in the 8-well group was mainly distributed in the proximal end of the screw. The bone cement in straight pore group was distributed evenly, and the bone cement surface was distributed in an annular manner along the entire length of the screw (Figs. 2 and 3). Furthermore, we performed CT imaging for each group and found that it was consistent with the X-ray results (Fig. 4).

## Discussions

In this study, enhancer-injecters with different number of holes were used to verify the effect of CPC injection on the augmentation of pedicle screw in the Chinese osteoporotic lumbar pedicle. we found that enhancer syringes with different hole numbers combined with CPC bone cement injection can significantly increase the maximum screw pull-out force. The 8-hole group has a smaller pull-out force and is relatively prone to leakage of reinforcing material, which lacks safety in use. The local reinforcement of 4-hole and 6-hole sheath can play a similar role to that of total nail tunnel reinforcement.

Furthermore, We thought that due to the change in the number of pores, the CPC distribution of 4-well, 6-well and 8-well bone cement is gradually distributed from the bottom to the top, which is related to local pressure. These also resulted in the 8-well syringe having less effect on the Maximum insertion torque and Maximum axial pull-out strength.

Pedicle screw fixation on osteoporotic vertebral bodies is easy to cause complications such as loosening and prolapse due to decreased bone density and bone mass. Loosening usually occurs at the interface between the screw and bone. It has been clinically verified that the maximum axial extraction force can be increased by injection of the reinforced material. Injection of polymethyl methacrylate (PMMA) bone cement can provide effective axial extraction force as a common method [7]. However, due to the release of a large amount of heat after mixing the powder with solvent, it is easy to cause thermal damage to peripheral nerves and blood vessels. In addition, PMMA is not biodegradable and may form a bioreactive film at the interface between the screw canal and bone, ultimately affecting the long-term stability of the screw canal [8]. Compared with PMMA, CPC has the characteristics of absorbability and bone induction [9]. Therefore, we conducted a study on the enhancement of pedicle screw channel under osteoporosis condition by using CPC, so as to add different bioactive materials to CPC as the research basis.

Our study found that the maximum torque value increased significantly after material perfusion, from 0.06 N·m to 0.12 N·m. This is closely related to the surrounding porous cancellous bone-like void structure filled by bone cement injection. However, because the amount of bone cement injected into the lateral pore sheath with different number of holes is similar, the distribution position is different, and the shape and scale of the inserted screws are the same, which are cylindrical screws rather than conical screws, so the influence on the maximum torque may not be very great. The insertion of the pedicle screw on the strengthened cancellous bone module is equivalent to the insertion of the screw into the bone with increased bone density, thus enhancing the maximum torque of the insertion [10].

The biomechanical test module used in this study is composed of polyurethane material. It has the characteristics of uniform material and uniform density, which can reduce the influence of bone density factors on the extraction force of pedicle screw and reduce the bias of experimental results, which has been recognized as a commercial mechanical test material in the world [11, 12]. It has been reported in the past that two kinds of polyurethane modules were used to test the torque and axial pulling force of three kinds of screws, and satisfactory results were obtained, which also confirmed the advantages of polyurethane material to simulate bone [13].

We found that the distribution of calcium phosphate cement around the screw showed a certain regularity after the juxtaposition of different lateral pore sheathes. The distribution of hardened bone cement blocks in the 4-holes, 6-holes and 8-holes groups was almost helical. The four holes were distributed in about 1/3 of the distal end of the screw, the six holes were distributed in about 1/3 of the middle part of the screw, and the eight holes were distributed in about 1/3 of the proximal end of the screw. According to the shape of the wrap around the pedicle screw after CPC injection, the wrap formed in the 8-holes group was roughly located at the screw head. In clinical application, bone cement overflow was likely to occur due to its proximity to the spinal canal. The main factors affecting the maximum axial pull-out strength are the shear strength between the material and the screw, the strength of the surrounding medium, the resistance formed by the proximal “bone” of the CPC along the axis of the screw after the wrapping of the screw and the material, and the shear force formed during the pulling out of the CPC bone cement and the surrounding medium [14, 15]. The injection of bone cement in the nail canal is equivalent to the injection of bone cement into the polyurethane material at different plane CPC in combination with the 4-, 6-, and 8-hole lateral empty sheath and the straight pore canal injection group. Firstly, the screw diameter is increased, while the straight pore canal injection increases the diameter along the full axial length of the screw, while the screw diameter in the control group does not change. Because the push-rod structure can basically ensure the same total amount of bone cement injected into the screw canal, the diameter of the package formed by injecting calcium phosphate bone cement through the straight pore canal is smaller than that of the bone cement strengthened through the side hole, which can be confirmed by X-ray examination. There was no statistical difference between the 6-holes group and the 4-holes group in the extraction force under osteoporosis conditions. Considering that the volume of injected bone cement was constant at 2.5ml, on the one hand, although the axial length of the bone cement wrap formed in the 6-well group was larger than that in the 4-holes group, the radius of the solidified wrap formed was slightly smaller than that in the 4-holes group. The axial pulling force of the screw depends on the resultant force between the shear force and the resistance above the cured wrap. The 6-holes group is located in the middle and lower part of the screw. Compared with the 4-holes group, although the medium resistance of solidified wrapping near the nail head will be reduced, the increase in its length and diameter may lead to the increase of the shear area in contact with the surrounding medium, resulting in a larger pulling force in the end. Due to the short distance between the wrapping and the nail head formed by the 8-hole group, the resistance from the upper medium in the pulling process is too small. Under the condition of the formation of a larger diameter, the overall drawing force of the 8-hole group is still the smallest. Although the wrapping diameter formed by the whole nail channel group is small, it is distributed along the full length of the nail channel, and the contact area with the surrounding medium polyurethane is large, so it can form a larger pull-out force.

## Conclusions

Enhancer syringes with different hole numbers combined with CPC bone cement injection can significantly increase the maximum screw pull-out force. The 8-hole group has a smaller pull-out force and is relatively prone to leakage of reinforcing material, which lacks safety in use. The local reinforcement of 4-hole and 6-hole sheath can play a similar role to that of total nail tunnel reinforcement.

## Data Availability

The raw data that support the findings of this study are available on request from the corresponding author, [Suochao Fu], upon reasonable request.

## References

[1] Kutsal FY, Ergin Ergani GO (2021). Vertebral compression fractures: still an unpredictable aspect of osteoporosis. Turk J Med Sci.

[2] Griffith JF (2015). Identifying osteoporotic vertebral fracture. Quant Imaging Med Surg.

[3] Ginebra MP, Canal C, Espanol M, Pastorino D, Montufar EB (2012). Calcium phosphate cements as drug delivery materials. Adv Drug Deliv Rev.

[4] Xu HH, Wang P, Wang L, Bao C, Chen Q, Weir MD (2017). Calcium phosphate cements for bone engineering and their biological properties. Bone Res.

[5] Wu T, Shi H, Liang Y, Lu T, Lin Z, Ye J (2020). Improving osteogenesis of calcium phosphate bone cement by incorporating with manganese doped beta-tricalcium phosphate. Mater Sci Eng C Mater Biol Appl.

[6] Fu S, Zhang Y, Ai F, Wang J, Wu Z, Ma X (2022). A novel bone cement injector augments Chinese osteoporotic lumbar pedicle screw channel: a biomechanical investigation. BMC Musculoskelet Disord.

[7] Kumar A, Ghosh R (2021). Fracture toughness of Acrylic PMMA Bone Cement: a Mini-review. Indian J Orthop.

[8] Bistolfi A, Ferracini R, Albanese C, Verne E, Miola M. PMMA-Based bone cements and the Problem of Joint Arthroplasty Infections: Status and New perspectives. Mater (Basel). 2019;12(23). 10.3390/ma12234002 PMC6926619 31810305

[9] Ji X, Xu F, Dong G, Jia C, Jia P, Chen H (2019). Loading necrostatin-1 composite bone cement inhibits necroptosis of bone tissue in rabbit. Regen Biomater.

[10] Meursinge Reynders RA, Ronchi L, Ladu L, van Etten-Jamaludin F, Bipat S (2012). Insertion torque and success of orthodontic mini-implants: a systematic review. Am J Orthod Dentofacial Orthop.

[11] Waits C, Burton D, McIff T (2009). Cement augmentation of pedicle screw fixation using novel cannulated cement insertion device. Spine (Phila Pa 1976).

[12] Hsu CC, Chao CK, Wang JL, Hou SM, Tsai YT, Lin J (2005). Increase of pullout strength of spinal pedicle screws with conical core: biomechanical tests and finite element analyses. J Orthop Res.

[13] Hashemi A, Bednar D, Ziada S (2009). Pullout strength of pedicle screws augmented with particulate calcium phosphate: an experimental study. Spine J.

[14] Makaram H, Swaminathan R (2021). Influence of bone quality and pedicle screw design on the fixation strength during Axial pull-out test: a 2D axisymmetric FE study. Annu Int Conf IEEE Eng Med Biol Soc.

[15] Salmoria KK, Tanaka OM, Guariza-Filho O, Camargo ES, de Souza LT, Maruo H (2008). Insertional torque and axial pull-out strength of mini-implants in mandibles of dogs. Am J Orthod Dentofacial Orthop.

